# Vascular Protective Effects of *Malus toringoides* (Rehd.) Hughes Extracts and their Mechanism in Diabetic Rats and HUVECs

**DOI:** 10.1155/2022/4348435

**Published:** 2022-10-19

**Authors:** Xiangyu Wang, Tong Wang, Liying Gao, Tsering Dikye, Linsha Dong, Ruiying Yuan, Ning Meng, Jing Yang, Eun-Rhan Woo, Dong-Sung Lee, Yuefei Wang, Bin Li, Shan Huang

**Affiliations:** ^1^Department of Pharmacy, Key Laboratory of Pharmaceutical Research for Metabolic Diseases, Qingdao University of Science & Technology, Qingdao 266042, China; ^2^University of Tibetan Medicine, Lhasa 850000, China; ^3^Key Laboratory of Combinatorial Biosynthesis and Drug Discovery (Wuhan University), Ministry of Education, Wuhan University School of Pharmaceutical Sciences, Wuhan 430071, China; ^4^Department of Pharmacy, College of Medicine, Tibet University, Lhasa 850000, China; ^5^Tianjin Key Laboratory of TCM Chemistry and Analysis, Tianjin University of Traditional Chinese Medicine, Tianjin 301617, China; ^6^College of Pharmacy, Chosun University, Dong-gu, Gwangju 61452, Republic of Korea

## Abstract

Malus toringoides (Rehd.) Hughes (Rosaceae) is used as a traditional folk medicine in the Tibet autonomous region of China to treat hypertension, hyperglycemia, and hyperlipidemia. However, few modern pharmacological data on the use of this plant against diabetic syndrome are available. In this study, we examined the vascular protection provided by a 70% ethanol extract of *M. toringoides* (EMT) in human umbilical vein endothelial cells (HUVECs) grown in high-glucose medium and in a high-fat diet/streptozotocin-induced rat diabetes model. EMT significantly suppressed the expression of cell adhesion molecules in both HUVECs and diabetic rats. EMT also inhibited activation of the CX3CL1/CX3CR1 axis and the nuclear factor kappa B (NF-*κ*B) signaling pathway *in vivo* and *in vitro*. The results provide a significant information on the vasoprotective properties of *M. toringoides* that may contribute to the development and application of related herbal medicines.

## 1. Background

Microvascular lesions of diabetes mellitus are responsible for complications, including diabetic nephropathy, diabetic cardiomyopathy among others. Macrovascular lesions can lead to hypertension, atherosclerosis, and peripheral arterial diseases [1, 2]. Of the many pathophysiological processes leading to diabetic angiopathy, the most important is vascular endothelial damage and dysfunction caused by persistent hyperglycemia. Endothelial cells are a natural barrier between circulating blood and vascular smooth muscle, aiding anticoagulation, vasodilation, and vascular development [3, 4]. Under pathological conditions, endothelial cells secrete a variety of adhesion molecules and chemokines, including intracellular adhesion molecule-1 (ICAM-1), vascular cell adhesion molecule-1 (VCAM-1), E-selectin, or monocyte chemotactic protein 1 (MCP-1), to participate in the inflammatory response [5, 6]. High-glucose stimulation leads to oxidative stress, endothelial dysfunction, increased secretion of adhesion molecules, promotes the adhesion, infiltration, and migration of lymphocytes and monocytes, and eventually leads to lipid accumulation and atherosclerosis [7–9].

In the cardiovascular system, the chemokine (C-X3-C motif) ligand 1 (CX3CL1)/chemokine (C-X3-C motif) receptor 1 (CX3CR1) axis not only directly affects cell migration and adhesion, but is also a signaling channel for CX3CL1 and other mediators [10–12]. CX3CL1 is a member of the CX3 chemokine family that exists in both soluble and membrane-bound forms [13, 14]. Soluble CX3CL1 induces the secretion of ICAM-1 and VCAM-1, which work together to stop leukocytes from multiplying. The complex formed by CX3CL1 binding to its receptor CX3CR1 on the surface of vascular endothelial cells activates CX3CL1/CX3CR1 axis signaling pathways that involve nuclear factor kappa B (NF-*κ*B), mitogen-activated protein kinase (MAPK), and phosphoinositide 3-kinase (PI3K) [15, 16].

Both insulin and oral medications effectively treat diabetes mellitus, but have side effects that include damage to the microvasculature, retina, and nerve tissue [17, 18]. Malus *toringoides* is a well-known folk medicine, and its use is described in “Tibetan Medicine the Crystal Mirror Materia Medica.” People drink *M. toringoides* preparations to alleviate indigestion, abdominal distention, and abdominal pain. Local Tibetan doctors prescribe water extracts and butter tea made with *M. toringoides* to cure hyperglycemia, hyperlipidemia, and liver disease. Phytochemical studies have revealed that the bioactive components of *M. toringoides* include flavonoids, fatty acids, and amino acids [19]. A lack of pharmacological and pharmacodynamic studies have made it difficult to determine the pharmaceutical potential of *M. toringoides* and its components. However, studies have shown that *M. toringoides* extracts have significant hypolipidemic and antioxidant effects in rats with high-fat diet-induced hyperlipidemia [20], and flavonoids in the plant have significant antidiabetes activity in mice and rats [21]. A high-fat diet and low-dose streptozotocin (HFD/STZ) induce type II diabetes in rats, and the pathogenesis is similar to that observed in human diabetes. The success rate of the rat model is high, and it is often accompanied by complications that include endothelial dysfunction [22, 23].

As a part of our ongoing research program, we evaluated the vascular protective effectiveness of a 70% ethanol extract of *M. toringoides* (EMT) by assaying adhesion molecules, oxidative stress, and inflammatory mediators in diabetic rats and high glucose-induced human umbilical vein endothelial cells (HUVECs).

## 2. Materials and Methods

### 2.1. Chemicals and Reagents

Low-glucose Dulbecco's modified Eagle medium (LG-DMEM, D6046), and other tissue culture reagents were from GIBCO BRL Co. (Grand Island, NY, USA). Total cholesterol (TC, A111-1-1), triglycerides (TG, A110-1-1), low density lipoprotein cholesterol (LDL-C, A113-1-1), high density lipoprotein cholesterol (HDL-C, A112-1-1), free fatty acids (FFAs, A042-1-1), catalase (CAT, A007-1-1), superoxide dismutase (SOD, A001-3-2), malondialdehyde (MDA, A003-1-2), and glutathione peroxidase (GSH-Px, A005-1-2) assay kits were obtained from Nanjing Jiancheng Bioengineering Institute (Nanjing, China). ELISA kits for C-reactive protein (CRP, EK0978) tumor necrosis factor alpha (TNF-*α*, EK0526), interleukin 1*β* (IL-1*β*, EK0393), IL-6 (EK0412), VCAM-1 (EK0537), ICAM-1 (EK0370), E-selectin (EK0501) and CX3CL1 (EK0356) were obtained from BOSTER Biological Technology (Wuhan, China). Primary antibodies including anti-p65 (ab32536), anti-CX3CL1 (ab25088), anti-CX3CR1 (ab245248), anti-ICAM-1 (ab282575), anti-VCAM-1 (ab134047) and anti-E-selectin (ab300557), anti-inhibitor of nuclear factor kappa B alpha (I*κ*B*α*) (ab32518), anti-phospho-I*κ*B*α* (ab133462), anti-lamin B (ab32535), anti-actin (ab179467), secondary antibodies such as goat anti-rabbit IgG (ab150077) and fluorescein isothiocyanate (FITC)-labelled secondary antibodies were from Abcam (Cambridge, UK). Reference standards for hyperoxide, isoquercitrin (A0439), quercitrin (A0084), phlorizin (A0216), phloretin (A0376), and oleanolic acid (A0117) were from MUST (Chengdu, China). Reagents for ultraperformance liquid chromatography-tandem mass spectrometry (UPLC-MS/MS) were purchased from Thermo Scientific (Waltham, MA, USA).

### 2.2. Plant Materials

Pulverized leaves of *M. toringoides* were extracted twice with 70% ethanol under reflux for 1.5 h per round. The resulting liquid was filtered and dried in vacuo to obtain the final product, which was submitted to the Component Bank of Tibetan Medicine in China (CBTM-E375).

### 2.3. Animal Experiments

Forty-eight male Sprague-Dawley rats were obtained from the Qingdao Institute for Food and Drug Control (approval number: SYXK Lu, 2019 0006) and housed in a room with a 12 h day: night cycle at a 22 ± 2°C. The animals were provided with an unlimited supply of food and drink. Diabetes model rats were prepared as previously described by Huang et al. [24] and divided into six groups of eight animals each: NC, (control); DM, (diabetes model); MET, (metformin, positive control, 250 mg/kg, intragastrical administration (i.g.)); L-EMT, (low-dose EMT, 195 mg/kg, i.g.); M-EMT (medium-dose EMT, 390 mg/kg, i.g.); and H-EMT (high-dose EMT, 780 mg/kg, i.g.). After 8 weeks of treatment, rats were sedated with sodium pentobarbital (Nembutal; 75 mg/kg body weight) and blood was drawn from the abdominal aorta. The animals were sacrificed by cervical dislocation. Half of the aortas were stored at −80°C and the remaining half were kept in formalin at 4°C until they were analyzed.

### 2.4. Biochemical Indices

Blood samples were taken and centrifuged for 15 minutes at 3,000 rpm to separate the serum, which was subsequently kept at −80°C until used. TG, TC, LDL-C, HDL-C, FFA, CAT, ROS, SOD, MDA, and GSH-Px concentration in serum or cells were assayed by appropriate kits. CRP, TNF-*α*, IL-1*β*, IL-6, VCAM-1, ICAM-1, E-selectin, and CX3CL1 levels were measured with ELISA kits.

### 2.5. Histological and Immunohistochemical Staining

Thoracic aortas were fixed with formalin for 24 h, washed with distilled water, dehydrated in ethanol gradient, and embedded in paraffin. The wax block was cut into 5 *μ*m serial sections using a microtome, mounted on glass slides and stained with hematoxylin and eosin. For immunohistochemical staining, the sections were dewaxed and 3% hydrogen peroxide (H_2_O_2_) was used to inactivate endogenous enzymes. Antigens were repaired by heating, blocking buffer was added for 30 min, and the mixture was incubated with anti-p65, anti-CX3CL1, anti-ICAM-1, anti-VCAM-1, or anti-E-selectin antibodies at 4°C overnight. Goat anti-mouse/rabbit immunoglobulin (Ig) G working solution was added for 30 min and a streptavidin-POD working solution was added with incubation at 37°C for 30 min. After washing in phosphate buffer solution, a diaminobenzidine solution was used for color development, and hematoxylin was used for counterstaining. The sections were observed by an optical microscope (CKX53, Olympus, Japan). Stained cells in random fields were statistically evaluated at a standard magnification. Image-Pro Plus 6.0 was used to quantify the imaging data.

### 2.6. Cell Culture and Viability Assay

HUVECs were obtained from the American Type Culture Collection (Manassas, VA, USA) and grown at 37°C and 5% CO_2_ in LG-DMEM containing 10% fetal bovine serum. Cell viability was evaluated with a methylthiazol tetrazolium (MTT) assay. Briefly, HUVECs were cultured in 96-well plates (5 × 10^4^ cells/mL) and incubated for 12 hours before adding EMT (50–800 *μ*g/mL) for 24 hours. Then, 50 *μ*L MTT (2.5 mg/mL) was added to each well. After 4 hours, the supernatant fraction was aspirated and 150 *µ*L dimethyl sulfoxide (DMSO) was added to each well. Absorbance was measured at 490 nm.

### 2.7. Western Blot Assays

Cells were lysed in RIPA lysis buffer containing phenylmethylsulfonyl fluoride (PMSF) and the protein concentration was tested with a Bradford assay kit. The proteins were mixed with loading buffer to adjust the cell homogenate concentration, and then loaded onto 10% sodium dodecyl sulfate (SDS)-polyacrylamide gels for electrophoresis and transferred to a nitrocellulose membrane. The membranes were incubated in blocking solution containing 5% nonfat dried milk for 2 h and then incubated with primary antibody including anti-p65 (1 : 1000), anti-CX3CL1 (1 : 1000), anti-CX3CR1 (1 : 1000), anti-ICAM-1 (1 : 1000), anti-VCAM-1 (1 : 1000), anti-E-selectin (1 : 1000), I*κ*B*α* (1 : 1000), anti-phospho-I*κ*B*α* (1 : 1000), anti-lamin B (1 : 1000), and anti-actin (1 : 1000) for 2 h. Then, the membrane was washed by TBST, and added with secondary goat anti-rabbit IgG (1 : 5000) for 1.5 h. The bands were observed with electrochemiluminescence (ECL) western blotting substrate. Protein intensity was measured by a ChemiDoc image analyzer (Tanon 4600, Tanon, China).

### 2.8. Extraction of Nuclear and Cytosolic Fractions

HUVEC cell nuclei and cytoplasm were extracted with a nucleoprotein extraction kit (Solarbio Technology Co., Ltd., Beijing, China). Briefly, HUVECs were collected, and to facilitate cell lysis, the cells were thoroughly mixed with the cytoplasmic extraction reagent and allowed to stand at 4°C for 10 min. The supernatant was the cytoplasmic extract. The precipitate was mixed well with the nuclear extraction reagent and incubated at 4°C for 15 min. The supernatant after centrifugation was the nuclear extract. The protein concentration of the cytoplasmic and nuclear extracts was determined with s bicinchoninic acid (BCA) assay kit.

### 2.9. Immunofluorescence Analysis

To identify NF-*κ*B or CX3CL1, HUVECs were inoculated on glass coverslips pre-treated with tissue culture reagents for 48 h and then fixed by adding paraformaldehyde to the coverslips for 30 min. The cells were permeabilized with TritonX-100 so that the NF-*κ*B or CX3CL1 antibodies could enter the cells. The cells were labelled with fluoresceine isothiocyanate (FITC)-labelled secondary antibodies and treated for 5 min with a 4′,6-diamidino-2-phenylindole (DAPI) solution to stain the nuclei. The slides were observed by fluorescence microscopy (CKX53, Olympus, Japan).

### 2.10. Statistical Analysis

The results were reported as means ± SD and the statistical analysis was performed with SPSS. One-way ANOVA was used to assess the difference between multiple groups, and *p* values <0.05 were considered significant.

## 3. Results

### 3.1. Effects of EMT on Glucose, Lipid Metabolism, And Antioxidant and Inflammation-Mediator Levels in Diabetic Rats

Changes in the body weight of the rats are shown in Table 1. Compared with the NC group, the mean weight in the diabetic group was reduced after STZ treatment (*P* < 0.01). After EMT treatment, body-weight loss was significantly increased in the H-EMT group (*P* < 0.01). As shown in Table 1, serum glucose levels were significantly increased (*P* < 0.01) in the DM group, and MET and EMT treatment were significantly decreased in serum glucose levels (*P* < 0.01). Besides, EMT treatment reduced serum lipid concentrations in rats with hyperlipidemia induced by a high-fat diet. STZ administration increased serum TC, TG, LDL-C, and FFA concentration in the DM group (*P* < 0.01) and decreased HDL-C levels (*P* < 0.01). After administration, LDL-C, TC, TG, and FFA were significantly lower and HDL-C was significantly higher in the EMT and MET treatment groups compared with the DM group.

**Figure 1:**
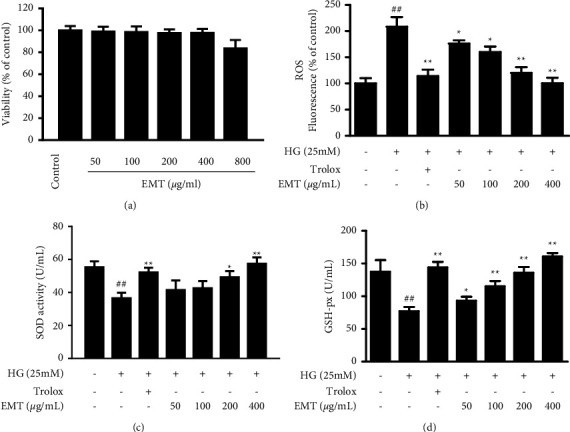
Effects of EMT on HUVECs viability and high glucose-induced ROS, SOD, and GSH-px levels in HUVECs. HUVECs were pretreated for 12 h with the indicated concentrations of EMT and then stimulated for 48 h with high glucose (25 mM). (a) Cells viability was evaluated by MTT assay. (b) Fluorescence probe DCFH-DA was used to detect ROS content in high glucose-stimulated HUVECSs for 30 min with protreatment of EMT. (c, d) Analyses of SOD and GSH-px levels were performed as described in the materials and methods section. Trolox (100 *μ*M) was used as the positive control. The values are mean ± SD of 3 independent experiments with triplicate samples. ^#^*P* < 0.05 or ^##^*P* < 0.01 compared to the control group. ^∗^*P* < 0.05 or ^∗∗^*P* < 0.01 compared to the group treated with high glucose.

The expression of antioxidants and inflammatory factors in serum were measured. As shown in Table 2, the activities of CAT, GSH-Px and SOD in the serum of the DM group decreased significantly (*P* < 0.01), and MDA, TNF-*α*, IL-6, IL-1*β*, and CRP expression increased (*P* < 0.01), indicating the occurrence of oxidative stress and inflammation. Figures 1(b)–1(d) shows that, compared with high glucose alone, EMT significantly inhibited the production of reactive oxygen species (*P* < 0.05) and upregulated SOD and GSH-Px activity in HUVECs (*P* < 0.01).

### 3.2. Effects of EMT on the Histopathology of the Aortas of Diabetic Rats

**Figure 2:**
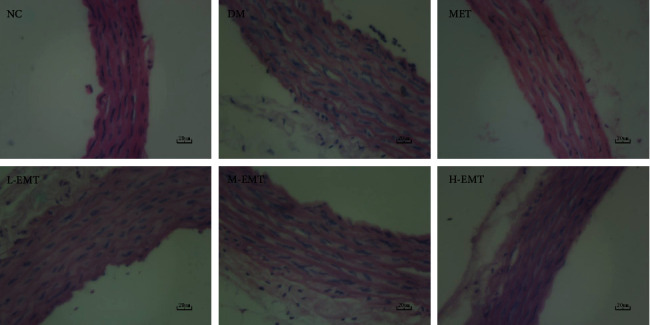
Effects of EMT on aortic histopathology in diabetic rats. Photomicrographs of aortic sections from a diabetic rat model established for 6 weeks and administered treatment for 8 weeks.

Figure 2 shows that in the NC group, endothelial cells were flat and the aortic intima was smooth. In contrast, the intima was rough and thickened in the DM group, and endothelial cell breakdown was present. The histological morphology of the aorta was clearly improved in the EMT group compared with the DM group.

### 3.3. Effects of EMT on the Expression of Adhesion Molecules in Diabetic Rats and in HUVECs

**Figure 3:**
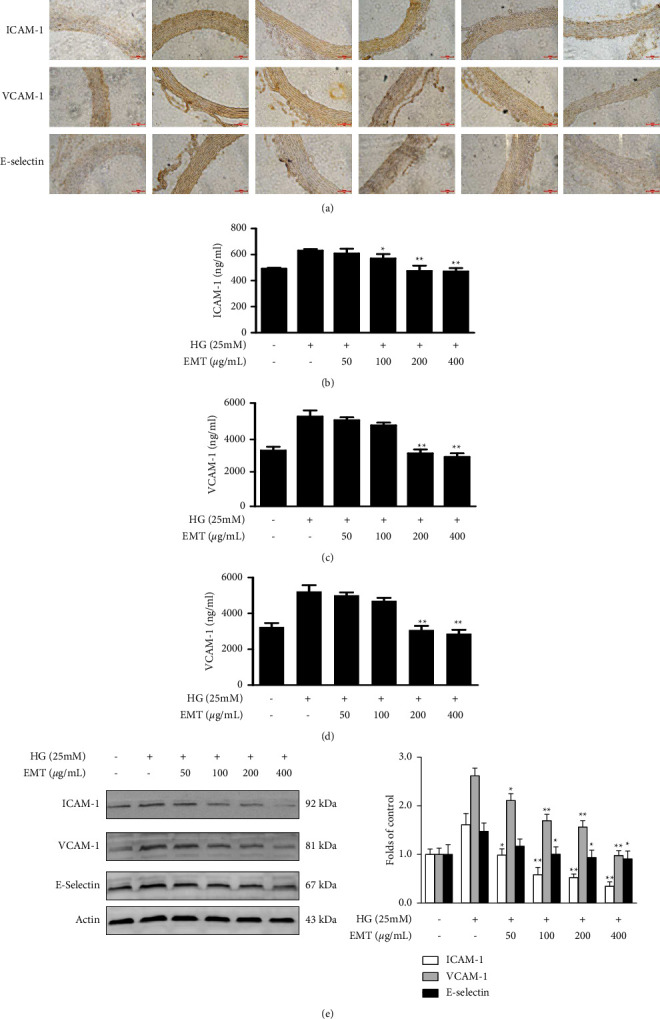
Effects of EMT on adhesion molecule expression in rat aortic histological sections and HUVECs. (a) Immunohistochemical analysis of adhesion molecule expression in aortic sections from a diabetic rat model established for 6 weeks and administered treatment for 8 weeks. (b–e) HUVECs were pretreated for 12 h with the indicated concentrations of EMT and stimulated for 48 h with high glucose (25 mM). ELISA and Western blot analyses of ICAM-1, VCAM-1, and E-selectin were performed as described in the materials and methods section. The values are mean ± SD of 3 independent experiments with triplicate samples. ^#^*P* < 0.05 or ^##^*P* < 0.01 compared to the group treated with high glucose.

The expression of adhesion molecules in aortic tissue was determined by immunohistochemistry. As shown in Figure 3(a) the concentrations of ICAM-1, VCAM-1, and E-selectin were higher in the in the DM group than in the NC group. However, their expression was significantly lower in the EMT treatment groups compared with the DM group. The histological changes were confirmed by *in vitro* analysis.

The cytotoxicity of EMT was measured by an MTT assay of the viability HUVECs in cell culture. Concentrations between 50 and 400 *µ*g/mL had no toxic effects in HUVECs (Figure 1(a)) and were used in subsequent cell culture experiments. The effects of EMT on the release of adhesion molecules by high glucose (25 mM)-induced HUVECs were determined by measuring ICAM-1, VCAM-1, and E-selectin concentration with quantitative ELISA kits. HUVECs were stimulated with high glucose for 24 h, Intracellular expression of the adhesion molecules increased significantly compared with the corresponding control values (*P* < 0.01). The results of both ELISA (Figures 3(b)–3(d)) and western blots (Figure 3(e)) showed that EMT pretreatment for 12 h suppressed the expression of adhesion molecules.

**Figure 4:**
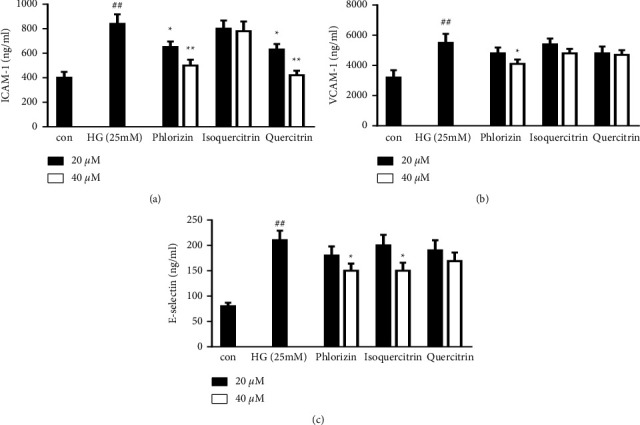
Effects of the EMT components phlorizin, isoquercitrin, and quercitrin on the expression of CAMs in high glucose-induced HUVECs. HUVECs were pretreated for 12 h with the indicated concentrations of phlorizin, isoquercitrin, and quercitrin and then stimulated for 48 h with high glucose (25 mM). ELISA analyses of ICAM-1, VCAM-1, and E-selectin were performed as described in the materials and methods section. The values are mean ± SD of 3 independent experiments with triplicate samples. ^#^*P* < 0.05 or ^##^*P* < 0.01 compared to the control group. ^∗^*P* < 0.05 or ^∗∗^*P* < 0.01 compared to the group treated with high glucose.

The main components of the EMT extract were identified by LC-MS/MS (Table S1 and Figure S1). To find the active components in EMT, we began by investigating the inhibitory effects of EMT components on the production of adhesion molecules in HUVECs in the *in vitro* system. As shown in Figure 4, phlorizin, isoquercitrin, and quercitrin selectively downregulated the production of ICAM-1, VCAM-1, and E-selectin, indicating that those EMT components may have vascular protective effects.

### 3.4. Effects of EMT on CX3CL1 Expression in Diabetic Rats and HUVECs

**Figure 5:**
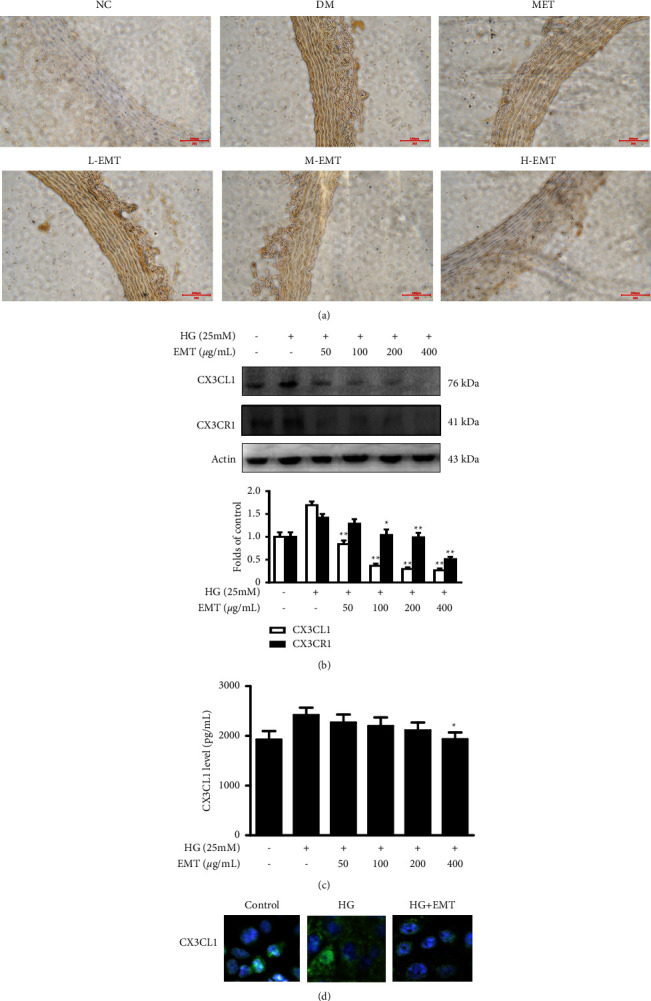
Effects of EMT on CX3CL1 expression in rat aortic histological sections and HUVECs. (a) Immunohistochemical detection of CX3CL1 expression in aortic sections from a diabetic rat model established for 6 weeks and administered treatment for 8 weeks. (b, c) HUVECs were pretreated for 12 h with the indicated concentrations of EMT and stimulated for 48 h with high glucose (25 mM). Western blot and ELISA analysis of CX3CL1 and CX3CR1 were performed as described in the materials and methods section. (d) After high glucose treatment, intense immunoreactions were observed in CX3CL1-positive cells (green), and blue staining of the nuclei was observed by DAPI. The values are mean ± SD of 3 independent experiments with triplicate samples. ^#^*P* < 0.05 or ^##^*P* < 0.01 compared to the group treated with high glucose.

CX3CL1 expression in the NC group and in the DM group were significantly different. After STZ administration, CX3CL1 levels significantly increased but returned to normal levels after 8 weeks of EMT administration (Figure 5(a)). Similar results were obtained in the *in vitro* experiments. Compared with the controls, high-glucose treatment resulted in significant increases in CX3CL1 and CX3CR1 protein expression (both *P* < 0.01). The increases in CX3CL1/CX3CR1 axis expression were significantly reduced by EMT at the tested concentrations (Figure 5(b)). The ELISA results showed that CX3CL1 was higher in HUVECs treated with high glucose than it was in the controls (*P* < 0.05). In the presence of EMT, high glucose-induced CX3CL1 expression was largely abrogated (Figure 5(c), *P* < 0.05). Immunofluorescence staining confirmed that high glucose induced a high percentage of CX3CL1-positive cells in HUVEC cultures and that EMT downregulated CX3CL1 expression (Figure 5(d)).

### 3.5. Effects of EMT on NF-*κ*B Activation in Diabetic Rats and HUVECs

**Figure 6:**
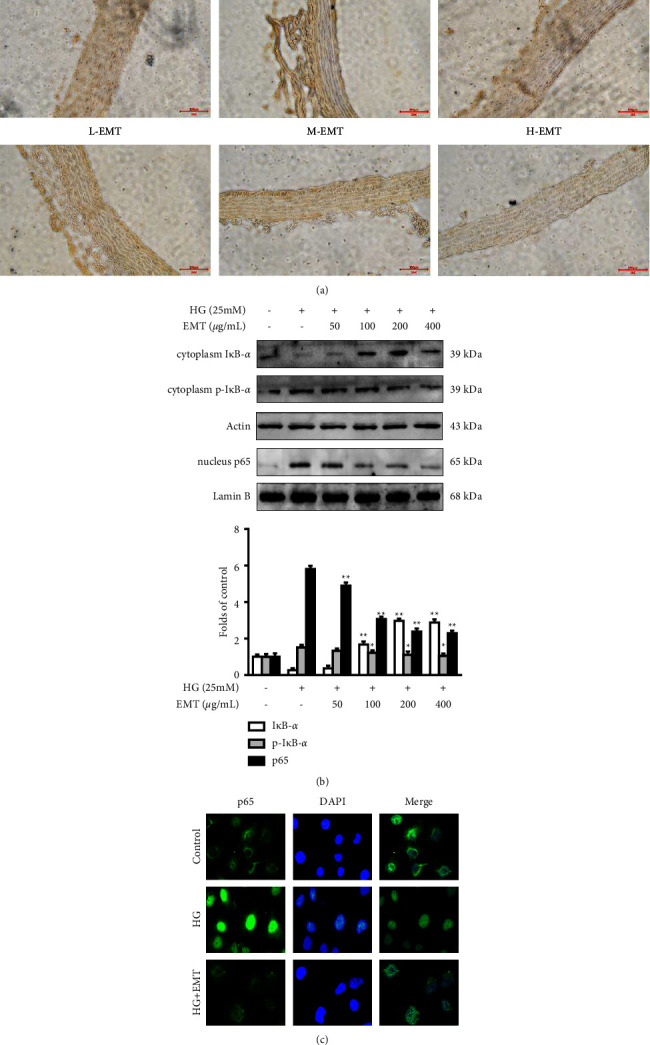
Effects of EMT on NF-*κ*B activation in rat aortic histological sections and HUVECs. (a) Immunohistochemical detection of NF-*κ*B activation in aortic sections from a diabetic rat model established for 6 weeks and administered treatment for 8 weeks. (b) Western blot analyses of I*κ*B-*α* and phospho-I*κ*B-*α* expression in the cytoplasm and NF-*κ*B p65 expression in the nucleus were performed as described in the materials and methods section, and representative blots of 3 independent experiments are shown. (c) NF-*κ*B p65 nuclear translocation was measured by immunofluorescence. The data represent the mean values of 3 experiments ± SD. ^#^*P* < 0.05 or ^##^*P* < 0.01 compared to the group treated with high glucose.

NF-*κ*B is related to the activation of specific target genes in HUVECs, including VCAM-1, ICAM-1, and others. Immunohistochemical analysis showed that NF-*κ*B was significantly upregulated in thoracic aorta samples from diabetic rats and that EMT downregulated NF-*κ*B expression (Figure 6(a)).  Figure 6(b) shows that EMT pretreatment (50–400 *µ*g/mL) for 12 h significantly inhibited the degradation of I*κ*B-*α* and reduced the nucleus p65 expression in HUVECs (*P* < 0.01). Notably, EMT significantly inhibited high glucose-induced activation of p65. The immunofluorescence results also showed that EMT inhibited p65 nuclear translocation (Figure 6(c)).

## 4. Discussion

The most authoritative publication on Tibetan medicine, “rGyud-bzhi,” contains a traditional description of the therapeutic effectiveness of *M. toringoides* in hypertension, dyspepsia, liver damage, hyperlipidemia, and hyperglycemia. In a previous study that evaluated the extraction and preparation of *M. toringoides* total flavonoids with a microporous adsorption resin, we found a high concentration of flavonoids with significant anti-inflammatory activity [25]. We also showed that *M. toringoides* had significant hypolipidemic and antioxidant activity in both rats with high-fat diet-induced hyperlipidemia and in HUVECs exposed to H_2_O_2_ [20]. The results of this study showed that EMT had significant hypoglycemic and hypolipidemic effects in a rat model of type II diabetes. As shown in Table 1, EMT significantly improved body-weight gain and glucose, TC, TG, HDL-c, LDL-c, and FFA levels in diabetic rats. It also significantly improved serum biochemical indicators of inflammation and oxidative stress in the diabetic rats. The results shown in Table 2 reveal that the levels of CAT and SOD were significantly increased by EMT, and GSH-Px, MDA, TNF-*α*, IL-6, IL-1*β*, and CRP expression were decreased. In subsequent histological observations, we found that EMT significantly improved vascular damage and dysfunction in diabetic rats. Figure 2 shows that EMT significantly improved the vascular morphology of diabetic rats.

The formation and progression of atherosclerosis are tightly correlated with the expression of cell adhesion molecules in atherosclerotic plaques [26, 27]. Previous studies have shown that VCAM-1, ICAM-1, and E-selectin are overexpressed on vascular endothelial cells in diabetes models, particularly in the early stages of atherosclerosis; E-selectin increases leukocyte rolling, while VCAM-1 and ICAM-1 induce leukocyte adhesion to endothelial cells and increase leukocyte migration to the endothelium [28, 29]. Figure 1 shows the protective effect of EMT on high glucose-induced HUVECs. Figure 3 shows that EMT decreased the expression of these adhesion molecules in the thoracic aorta of diabetic rats. EMT also inhibited the expression of ICAMs in high glucose-induced HUVECs. We also evaluated the inhibition of the main components of EMT of the production of adhesion molecules in an in vitro system. The results indicated that the vascular protection of EMT may have been mediated by phlorizin, isoquercitrin, and quercitrin to some extent (Figure 4). Our ongoing research aims to further evaluate and determine the pharmacological activity and mechanisms of action of these components to determine the pharmacodynamic basis of the vascular protection provided by EMT.

CX3CL1 is a unique chemokine with chemoattractant and adhesive properties. It is overexpressed throughout the development of atherosclerosis and is involved in endothelial cell dysfunction and atherosclerotic plaque formation [30, 31]. Under pathological conditions, the binding of CX3CL1 released by endothelial cells to the CX3CR1 receptor on natural killer cells enhances their killing activity and results in endothelial cell damage [32, 33]. CX3CL1 also promotes excess secretion of cell adhesion molecules and chemokines that promote adhesion to and infiltration of monocytes to the vascular endothelium and accelerate the development of atherosclerosis [34, 35]. The study results showed that hyperglycemia-induced CX3CL1 overexpression was significantly reduced by EMT both *in vivo* and *in vitro* (Figure 5).

In addition to being directly involved in the pathological process, CX3CL1 also activates transcription through the NF-*κ*B, PI3K, MAPK and other signaling pathways to regulate the expression of genes that code for adhesion molecules and inflammatory mediators [36, 37]. Mussbacher et al. [38] reported that the transcription factor NF-*κ*B, which selectively binds to distinct gene promoters to regulate the expression of multiple cellular genes, is involved in the development of atherosclerosis. When activated, I*κ*B-*α* is quickly phosphorylated, and p65 is transferred to the nucleus where it activates the transcription of genes, such as VCAM-1 and E-selectin [39, 40].  Figure 6 illustrates the events involved in the inhibition of p65 expression by EMT treatment.

## 5. Conclusion

EMT, a 70% ethanol extract of *M. toringoides*, significantly improved biochemical indicators of inflammation and oxidative stress in the serum of diabetic rats. The potent vasoprotective effects of this plant may occur through downregulation of the CX3CL1/CX3CR1 axis and expression of NF-*κ*B-dependent endothelial adhesion molecules. Our results provide information on the vasoprotective properties of *M. toringoides* that may contribute to the development and application of related herbal medicines. Follow-up studies are planned to evaluate the pharmacokinetics and quality standards of the active components of EMT to provide a better theoretical foundation for the use of *M. toringoides*-related products.

## Figures and Tables

**Table 1:** Effects of EMT on body weight and serum levels of glucose and lipid profile in diabetic rats.

	NC	DM	MET	L-EMT	M-EMT	H-EMT
Body weight (g)	370.50 ± 18.80	214.14 ± 11.37^##^	238.17 ± 26.47^∗^	220.26 ± 14.46	230.75 ± 19.27	242.50 ± 17.85^∗∗^
Glucose (mmol/L)	5.42 ± 0.59	25.61 ± 2.24^##^	11.27 ± 0.48^∗∗^	15.15 ± 2.37^∗∗^	13.18 ± 1.71^∗∗^	11.03 ± 2.29^∗∗^
TC (mmol/L)	0.25 ± 0.07	0.48 ± 0.01^##^	0.30 ± 0.05^∗∗^	0.35 ± 0.04^∗∗^	0.33 ± 0.07^∗∗^	0.25 ± 0.04^∗∗^
TG (mmol/L)	0.33 ± 0.15	0.68 ± 0.12^##^	0.42 ± 0.08^∗∗^	0.47 ± 0.10^∗∗^	0.40 ± 0.06^∗∗^	0.36 ± 0.04^∗∗^
HDL-C (mmol/L)	6.34 ± 0.30	2.88 ± 0.11^##^	12.25 ± 0.83^∗∗^	3.08 ± 0.17^∗^	4.02 ± 0.33^∗∗^	4.65 ± 0.12^∗∗^
LDL-C (mmol/L)	12.86 ± 0.11	15.36 ± 0.86^##^	12.90 ± 0.68^∗∗^	14.34 ± 0.24^∗∗^	13.64 ± 0.12^∗∗^	13.40 ± 0.31^∗∗^
FFA (*μ*mol/L)	4909.09 ± 735.62	12818.64 ± 792.24^##^	7704.55 ± 60.66^∗∗^	10863.64 ± 851.2^∗∗^	9000.08 ± 363.9^∗∗^	5954.55 ± 424.62^∗∗^

MET (100 *μ*M) was used as the positive control. Results are mean ± SD, *n* = 8. ^#^*P* < 0.05 vs. Con group, ^##^*P* < 0.01 vs. Con group, ^∗^*P* < 0.05 vs. DM group, ^∗∗^*P* < 0.01 vs. DM group.

**Table 2:** Effects of EMT on oxidative stress and inflammatory mediators levels in diabetic rats.

	NC	DM	MET	L-EMT	M-EMT	H-EMT
CAT (U/mL)	16.71 ± 1.29	3.80 ± 0.44^##^	13.20 ± 1.12^∗∗^	4.02 ± 0.38	7.81 ± 0.70^∗∗^	16.20 ± 1.13^∗∗^
MDA (nmol/mL)	22.80 ± 2.44	42.46 ± 2.87^##^	30.98 ± 2.37^∗∗^	27.77 ± 1.74^∗∗^	26.37 ± 1.34^∗∗^	16.49 ± 1.69^∗∗^
GSH-px (U/mL)	277.04 ± 14.95	220.74 ± 13.196^##^	275.18 ± 15.18^∗^	272.59 ± 14.14	298.52 ± 13.24^∗∗^	306.67 ± 16.66^∗∗^
SOD (U/mL)	14.95 ± 1.19	10.80 ± 1.82^##^	12.89 ± 0.62^∗∗^	11.26 ± 1.63	14.07 ± 0.84^∗∗^	15.00 ± 1.26^∗∗^
TNF-*α* (ng/mL)	77.07 ± 5.00	126.18 ± 6.62^##^	89.33 ± 7.03^∗∗^	108.20 ± 7.00*∗*	92.29 ± 4.26^∗∗^	38.77 ± 2.25^∗∗^
IL-6 (pg/mL)	43.77 ± 3.07	109.56 ± 7.82^##^	64.47 ± 3.37^∗∗^	80.00 ± 5.70^∗∗^	44.07 ± 2.09^∗∗^	35.53 ± 0.74^∗∗^
IL-1*β* (ng/mL)	7.81 ± 0.34	19.53 ± 2.11^##^	9.92 ± 0.67^∗∗^	15.31 ± 1.37^∗∗^	8.49 ± 0.76^∗∗^	6.88 ± 0.76^∗∗^
CRP (*μ*g/mL)	305.63 ± 11.50	365.87 ± 11.58^##^	306.66 ± 14.00^∗∗^	386.54 ± 13.50^∗∗^	324.35 ± 14.30^∗∗^	276.03 ± 12.62^∗∗^

Results are mean ± SD, *n* = 8. ^#^*P* < 0.05 vs. Con group, ^##^*P* < 0.01 vs. Con group, ^∗^*P* < 0.05 vs. DM group, ^∗∗^*P* < 0.01 vs. DM group.

## Data Availability

The datasets used and/or analyzed during the current study are available from the corresponding author on reasonable request.
