# Supplementary figures and images for: Vascular Protective Effects of Malus toringoides (Rehd.) Hughes Extracts and their Mechanism in Diabetic Rats and HUVECs
Source: Evid Based Complement Alternat Med. 2022 Oct 19;2022:4348435. doi: 10.1155/2022/4348435 (PMC9605824; doi:10.1155/2022/4348435)

## Slide 1
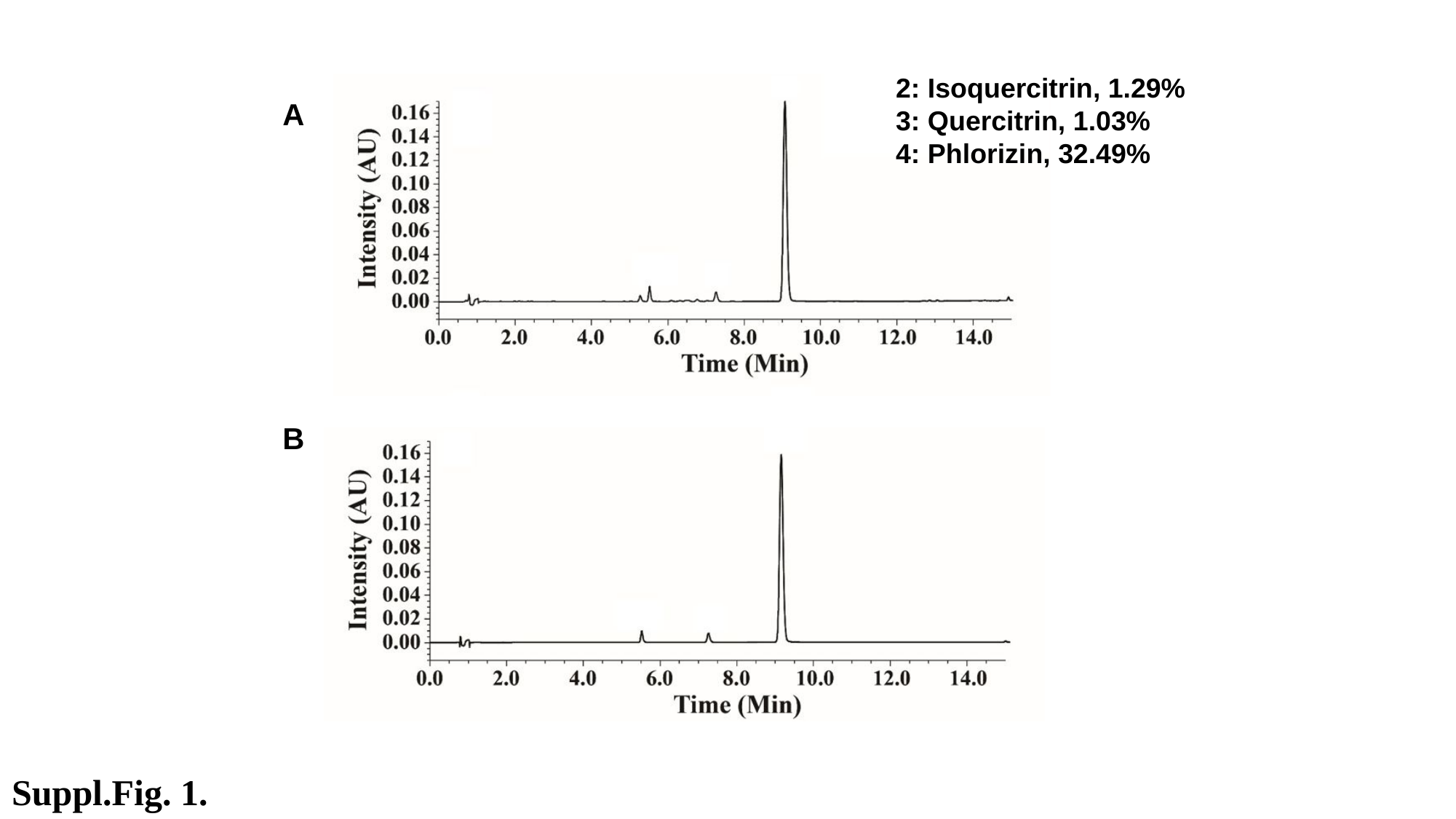

2: Isoquercitrin, 1.29%
3: Quercitrin, 1.03%
4: Phlorizin, 32.49%
A
B
Suppl.Fig. 1.

Supplement: Supplementary Materials — Supplementary data to this article can be found in supplemental files. [file 4348435.f1.pptx]
